# Supplementary material for: Mechanisms governing the pioneering and redistribution capabilities of the non-classical pioneer PU.1
Source: Nat Commun. 2020 Jan 21;11:402. doi: 10.1038/s41467-019-13960-2 (PMC6972792; doi:10.1038/s41467-019-13960-2)
Supplement: Supplementary file 7 — Source data [file 41467_2019_13960_MOESM7_ESM.zip › Source_Data/Figure6/Figure6B_MotifScanOutput/ETS1_PU.1/homerResults/motif12.similar.html]

motif12

## Information for motif12

T
G
C
A
A
T
G
C
G
A
C
T
T
C
G
A
G
A
T
C
T
G
C
A
G
C
T
A
A
G
C
T
G
A
C
T
G
A
T
C
A
G
T
C
G
T
A
C
  
Reverse Opposite:  

C
A
T
G
C
T
A
G
C
T
A
G
C
T
G
A
T
C
G
A
C
G
A
T
A
C
G
T
C
A
T
G
A
G
C
T
C
T
G
A
T
A
C
G
A
C
G
T
  

|  |  |
| --- | --- |
| p-value: | 1e-171 |
| log p-value: | -3.940e+02 |
| Information Content per bp: | 1.661 |
| Number of Target Sequences with motif | 771.0 |
| Percentage of Target Sequences with motif | 4.87% |
| Number of Background Sequences with motif | 254.8 |
| Percentage of Background Sequences with motif | 0.79% |
| Average Position of motif in Targets | 102.2 +/- 47.7bp |
| Average Position of motif in Background | 96.2 +/- 68.1bp |
| Strand Bias (log2 ratio + to - strand density) | 0.1 |
| Multiplicity (# of sites on avg that occur together) | 1.26 |
| Motif File: | file (matrix) reverse opposite |

### Similar de novo motifs found

|  |  |  |  |  |  |  |  |
| --- | --- | --- | --- | --- | --- | --- | --- |
| Rank | Match Score | Redundant Motif | P-value | log P-value | % of Targets | % of Background | Motif file |
| 1 | 0.959 | T G C A G T A C A G C T T C G A A G T C C T G A G C T A A G C T G A C T A G T C A G T C G T A C C T G A | 1e-165 | -381.668788 | 3.74% | 0.40% | motif file (matrix) |
| 2 | 0.945 | T G C A G T A C G A C T T G C A A G T C T C G A C G T A A G C T G A C T A G T C A G T C G A T C C T G A C A T G | 1e-155 | -357.763189 | 3.75% | 0.46% | motif file (matrix) |
| 3 | 0.913 | G T C A T A G C A G C T T G C A A G T C T C G A G C T A A G C T C G A T A G T C G A T C | 1e-142 | -327.727980 | 4.23% | 0.74% | motif file (matrix) |
| 4 | 0.812 | C G T A A G T C A C G T C G T A A G T C C T G A T C G A C A G T A C G T A G T C | 1e-90 | -209.288406 | 3.30% | 0.74% | motif file (matrix) |
